# The effect of beverages and cleansers on the color stability and surface roughness of conventional and digital denture base resins

**DOI:** 10.55730/1300-0144.6084

**Published:** 2025-09-15

**Authors:** Zeynep ŞAHİN, Nazire Esra ÖZER, Mehmet Ali KILIÇARSLAN

**Affiliations:** 1 Department of Prosthodontics, Faculty of Dentistry, Lokman Hekim University, Ankara, Turkiye; 2 Department of Prosthodontics, Faculty of Dentistry, Ankara University, Ankara, Turkiye

**Keywords:** CAD/CAM, denture base, color, denture cleanser, surface properties

## Abstract

**Background/aim:**

Research on the optical and surface properties of 3D-printed denture base materials is limited. This study investigated the effects of different beverages and denture cleansers on the color stability and surface roughness of conventional and digital denture bases.

**Materials and methods:**

A total of 240 specimens were prepared using two digital (CAD/CAM milling and 3D printing) and two conventional (cold- and heat-polymerized) materials. The specimens were immersed in tea, coffee, and artificial saliva for 12 days. After immersion, specimens were cleaned with either sodium hypochlorite or a Corega effervescent tablet. Color changes and surface roughness were measured with a colorimeter and a profilometer, respectively. Statistical analyses were performed with ANOVA, t-tests, Kruskal–Wallis, and Wilcoxon tests depending on data distribution.

**Results:**

3D-printed resins exhibited greater discoloration in tea and coffee than in artificial saliva, whereas heat-polymerized and CAD/CAM-milled resins were more color stable. Corega generally reduced discoloration more effectively than NaOCl. Surface roughness increased significantly in 3D-printed and cold-polymerized resins after staining and selected cleansing procedures (p < 0.05), whereas heat-polymerized and CAD/CAM-milled materials maintained lower roughness values.

**Conclusion:**

All materials showed clinically acceptable color changes (ΔE_00_ ≤ 4.1). Heat-polymerized and CAD/CAM-milled resins demonstrated superior surface integrity, suggesting a lower risk of plaque accumulation. Cold-polymerized and 3D-printed resins were more susceptible to staining and increased roughness, underscoring the importance of careful material selection and customized hygiene recommendations to preserve denture esthetics and longevity.

## 1. Introduction

Edentulism is a common health condition that negatively affects the oral health and quality of life of patients, particularly elderly individuals rehabilitated with conventional or implant-supported prostheses [1]. Two main methods are used to manufacture prostheses: conventional fabrication and computer-aided design and manufacturing (CAD/CAM) [2]. A variety of materials can be used to fabricate dentures [3]. Polymethyl methacrylate (PMMA) is widely used for denture bases because it is inexpensive, biocompatible, easy to fabricate and repair, and esthetically pleasing [4–6]. However, PMMA is prone to dimensional changes, fractures, color instability, surface abrasion, tissue irritation, and microbial colonization. Surface irregularities and subsurface porosities can compromise the mechanical properties and the esthetic and hygienic outcomes of dentures [7].

Digital denture fabrication offers improved precision and mechanical properties, reduces chairside time, and eliminates the need for conventional impressions [2]. Digital prostheses can be produced via subtractive (milling) or additive (3D printing) techniques [4]. Comparative studies of 3D-printed and conventional denture base resins have shown that 3D-printed resins exhibit better color stability and fracture toughness. CAD/CAM-milled resins have been reported to possess superior surface characteristics, including surface roughness, gloss, flexural strength, and elastic modulus [1].

Color stability and surface roughness are key factors that determine denture longevity. Increased surface roughness facilitates microbial colonization and staining, negatively affecting esthetics and patient satisfaction [8,9]. Exposure to staining beverages (e.g., coffee, tea, red wine) and routine hygiene procedures further influence these properties [7,10–14]. Although mechanical brushing is generally effective, elderly patients rely on chemical denture cleansers, such as sodium hypochlorite (NaOCl) or effervescent tablets, due to limited manual dexterity [15–17]. While these agents effectively remove stains and control microbial growth, they may also cause color changes and surface degradation, thereby compromising both esthetic and mechanical performance [7,8,12,16–19].

Recent systematic reviews and meta analyses have evaluated the separate effects of staining beverages, cleansing agents, and mechanical challenges on the color stability, surface properties, and overall durability of denture base materials, including both conventional and digitally fabricated resins [20,21]. For example, Sessa et al. [20] evaluated the effects of staining beverages and cleansing agents on the color stability of heat-polymerized and CAD/CAM resins, whereas Khan et al. [21] examined the effects of denture cleansers on the surface properties of conventional and digitally fabricated bases. Another review investigated the impact of simulated toothbrushing with dentifrices on the surface roughness and mass loss of acrylic resins [6]. Collectively, these studies highlight that both esthetic and mechanical performance can be affected in conventional, CAD/CAM-milled, and 3D-printed denture bases. However, none of these studies have investigated the combined effects of staining and cleansing protocols on PMMA denture bases, which would more accurately simulate clinical conditions.

This knowledge gap is particularly relevant to different fabrication methods, including conventional (cold- and heat-polymerized), CAD/CAM-milled, and 3D-printed bases. Understanding these combined effects is crucial for guiding material selection and maintenance strategies to ensure long-term esthetic and functional outcomes.

Based on this rationale, the null hypotheses of the present study were as follows:

1. There is no significant difference in color change (ΔE_00_) or surface roughness (Ra) between digital and conventional denture bases after exposure to various beverages.
2. The is no significant difference in the effects of different denture cleansers on the color change and surface roughness of denture bases.

## 2. Materials and methods

### 2.1. Study design and sample size

This in vitro study investigated the effects of sequential staining and cleaning on the color stability and surface roughness of denture base resins under standardized laboratory conditions. Detailed information on the denture base materials is presented in Table 1, and the devices and instruments used in the study are summarized in Table 2.

Sample size estimation was performed using G*Power (version 3.1.9.4; Heinrich Heine University, Düsseldorf, Germany), assuming a comparison involving 24 groups. The results indicated that a minimum of 240 specimens (10 per subgroup) was required, considering an alpha level of 0.05, a power of 95%, and an effect size of 0.40.

### 2.2. Specimen preparation

Digital specimens were designed using CAD software (SolidWorks; Dassault Systemes SolidWorks Corp., Waltham, MA, USA) and converted to standard tessellation language (STL) format. The milling group specimens were prepared using a milling device, whereas those in the 3D printing group were produced with a 3D printer and subjected to post-polymerization according to the manufacturer’s instructions. The conventionally fabricated specimens were obtained by milling wax models of identical dimensions. After flasking, wax elimination was performed, followed by application of a separating medium. Heat-cured specimens were prepared according to the manufacturer’s instructions, using a prolonged curing cycle (74 °C for 9 h) with the conventional compression molding technique. For cold-cured specimens, negative spaces were created, the acrylic resin was mixed, and polymerization was performed at room temperature for 2 min, followed by 10 min at 55 °C under 2 bar pressure in a pressure-curing pot. After polymerization, all specimens were deflasked, finished, and their dimensions were verified with calipers. Subsequently, all prepared specimens were trimmed and smoothed with 400-, 600-, and 800-grit water sandpapers to ensure standardization, followed by fine polishing with a polishing paste. The specimens were then subjected to ultrasonic cleaning in distilled water for 10 min and dried with a paper towel before baseline measurements (t1).

### 2.3. Staining protocol

Specimens were randomly allocated into three groups for immersion in tea, coffee, or artificial saliva (Table 3) and stored in a dark chamber until the experiment. Artificial saliva was prepared in-house using a standardized composition of salts and urea, adjusted to pH 6.5. Coffee and tea solutions were freshly prepared daily. Specimens were immersed in the respective solutions for 288 h, with the solutions replaced every 24 h, simulating approximately 1 year of clinical exposure. After immersion, specimens were rinsed with distilled water, dried, and color and surface roughness were remeasured (t2).

### 2.4. Cleaning protocol

Each group was further divided into two subgroups for cleaning with either an effervescent tablet or 1% sodium hypochlorite solution (Table 3). To simulate 180 days of denture cleaning, nine cleaning cycles were performed over 20 days [18]. After each cycle, specimens were rinsed under running water for 30 s [22]. Fresh cleaning solutions were prepared for each cycle. Specimens in the effervescent tablet group were immersed for 3 min, whereas those in the hypochlorite group were immersed for 10 min at room temperature. After all cycles, specimens underwent ultrasonic cleaning for 15 min and were air-dried before final measurements (t3).

### 2.5. Color and surface roughness measurements

Color and surface roughness were assessed at three time points: baseline (t1), after staining (t2), and after cleaning (t3). Color was measured with a colorimeter, and surface roughness was measured with a contact profilometer (Table 2). All measurements were performed by the same operator under standardized laboratory conditions.

Color differences (ΔE_00_) were calculated using the CIEDE2000 formula:


$$\Delta {\text{E}}_{00}={\left[{\left(\frac{\Delta L^{\prime }}{k_{L}\;s_{L}}\right)}^{2}+{\left(\frac{\Delta C^{\prime }}{k_{C}\;s_{C}}\right)}^{2}+{\left(\frac{\Delta H^{\prime }}{k_{H}\;s_{H}}\right)}^{2}+R_{T}(\frac{\Delta C^{\prime }}{k_{C}\;s_{C}})\times (\frac{\Delta H^{\prime }}{k_{H}\;s_{H}})\right]}^{1/2}$$

In this equation, ΔL′, ΔC′, and ΔH′ represent changes in lightness, chroma, and hue, corrected using weighting factors (SL, SC, SH) and parametric constants (KL, KC, KH), all set to 1 [9]. Measurements were based on the CIELAB color space, where L* indicates lightness, a* represents the red–green axis, and b* represents the blue–yellow axis [10].

For clinical interpretation, perceptibility and acceptability thresholds were applied using the 50:50 criteria: ΔE_00_ ≤ 1.7 was considered imperceptible, values between 1.7 and 4.1 were clinically acceptable, and values above 4.1 were deemed unacceptable [11,23].

### 2.6. Statistical analysis

All statistical analyses were performed using SPSS version 22 (IBM Corp., Armonk, NY, USA) and GraphPad Prism (GraphPad Sofware Inc., San Diego, CA, USA). Descriptive data were presented as means with standard deviations or medians with interquartile ranges, as appropriate. Data normality was assessed using the Shapiro–Wilk test, and homogeneity of variance was evaluated with Levene’s test. Parametric tests, including t-tests and one-way ANOVA, were applied to normally distributed datasets, whereas nonparametric tests such as Wilcoxon, Mann–Whitney U, and Kruskal–Wallis were used for nonnormal data. Statistical significance was set at p < 0.05.

## 3. Results

Most ΔE_00_ and Ra measurements did not follow a normal distribution (Shapiro–Wilk, p < 0.05) and were therefore analyzed using nonparametric statistical methods. Exceptions included ΔE_00_ values in the tea + Corega and coffee + Corega subgroups and Ra values for solution comparisons in the heat-polymerized group after Corega cleaning, which satisfied normality and homogeneity assumptions (Shapiro–Wilk and Levene’s, p > 0.05) and were subsequently analyzed using one-way ANOVA with Tukey’s post hoc test. For cleaning agent comparisons (Corega versus NaOCl), independent t-tests were applied to normally distributed subgroups (heat-polymerized + coffee, CAD/CAM + tea, 3D-printed + tea, 3D-printed + coffee), whereas nonparametric subgroups were analyzed using the Mann–Whitney U test.

ΔE_00_ values and statistical comparisons after immersion in artificial saliva, tea, and coffee are summarized in Table 4. Immersion in artificial saliva caused greater discoloration in cold-polymerized and CAD/CAM-milled materials compared with heat-polymerized and 3D-printed materials (p < 0.05). After tea immersion, the heat-polymerized group showed the least color change, whereas 3D-printed specimens exhibited greater discoloration than CAD/CAM-milled specimens (p < 0.05). In coffee, heat-polymerized and 3D-printed materials showed significantly greater color change than cold-polymerized specimens, with the heat-polymerized group also exhibiting more discoloration than CAD/CAM-milled specimens; no significant difference was found between CAD/CAM-milled and 3D-printed materials (p > 0.05).

Intramaterial comparisons showed that cold-polymerized specimens exhibited greater discoloration in artificial saliva and tea than in coffee. Heat-polymerized specimens showed greater color change in coffee than in tea or artificial saliva. CAD/CAM specimens were most affected by artificial saliva, whereas tea and coffee caused greater discoloration in 3D-printed materials.

**Figure 1:**
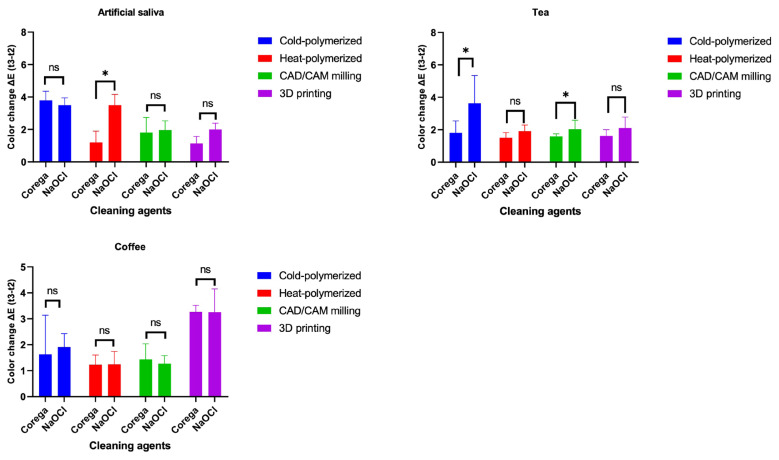
Comparison of color changes (ΔE_00_) in test materials after staining and subsequent cleaning with different agents. * p < 0.05 indicates a statistically significant difference between NaOCl and Corega.

For cold-polymerized materials, no significant differences were found between Corega and NaOCl in artificial saliva or coffee. In tea, Corega caused significantly less discoloration than NaOCl (p < 0.001). In the heat-polymerized group immersed in artificial saliva, Corega preserved color stability of the specimens better than NaOCl (p = 0.035). In the CAD/CAM group, Corega was more effective than NaOCl in the tea subgroup (p = 0.001), whereas no significant differences were observed in the other solutions. The 3D-printed group showed no cleaning agent-related differences in any solution (p > 0.05; Figure 1).

Comparisons within the same staining–cleaning subgroup showed that Corega-treated cold-polymerized specimens in artificial saliva exhibited significantly greater color change than other materials (p < 0.001). No significant differences were observed in the tea + Corega subgroup. In the coffee + Corega group, 3D-printed materials exhibited the highest color change (p < 0.001). NaOCl-treated cold-polymerized specimens in artificial saliva and tea exhibited greater color change than the CAD/CAM and 3D-printed groups. Similarly, in the coffee + NaOCl subgroup, 3D-printed materials exhibited greater discoloration than the heat-polymerized and CAD/CAM groups (Table 5).

All Ra values are presented in Table 6. At baseline, 3D-printed materials showed the highest Ra values, followed by cold-polymerized specimens, whereas heat-polymerized and CAD/CAM-milled materials exhibited the lowest roughness (p < 0.001). Immersion in artificial saliva, tea, or coffee did not significantly alter roughness in most groups (p > 0.05). However, in the heat-polymerized + Corega subgroup, coffee immersion significantly increased Ra compared with artificial saliva (p = 0.021). No such difference was observed in the heat-polymerized + NaOCl subgroup.

**Figure 2:**
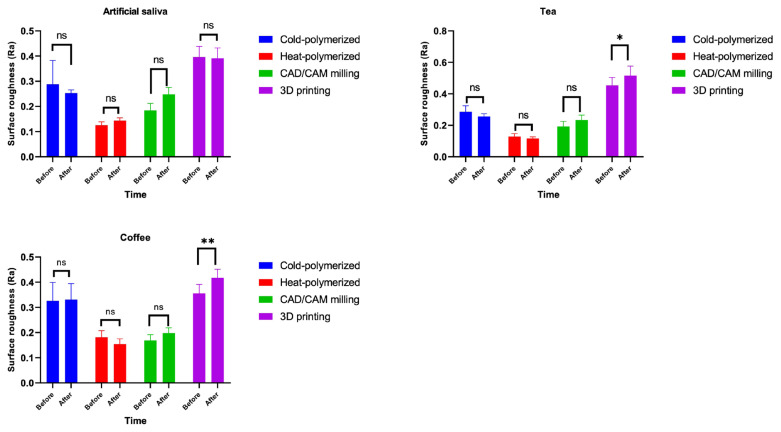
Surface roughness (Ra) of test materials at baseline and after immersion in artificial saliva, tea, and coffee. * p < 0.05 indicates a statistically significant difference between baseline and poststaining measurements.

**Figure 3:**
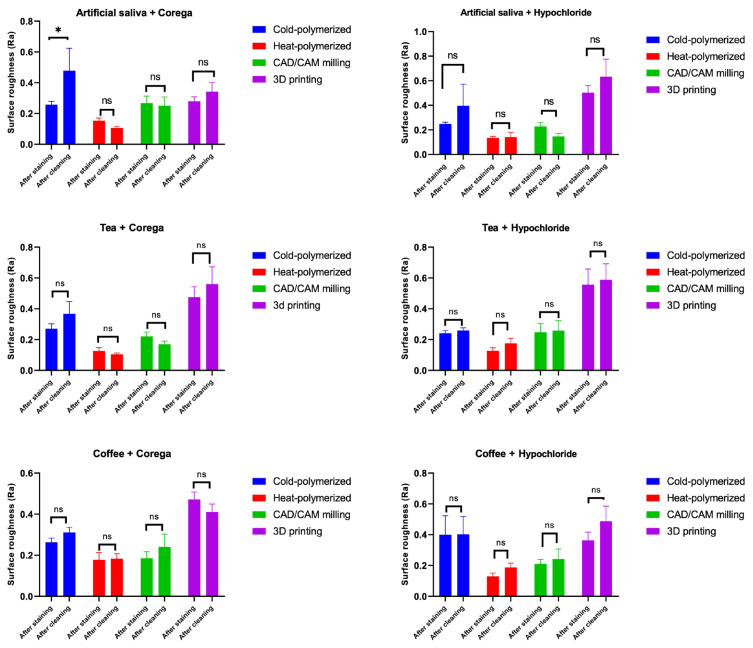
Surface roughness (Ra) of test materials after staining and subsequent cleaning with Corega tablets or sodium hypochlorite (NaOCl). * p < 0.05 indicates a statistically significant difference between poststaining and postcleaning measurements.

Postcleaning comparisons revealed that NaOCl increased surface roughness in 3D-printed materials more than Corega (p = 0.026), whereas no significant differences were found in the other materials (p > 0.05; Table 6). Significant Ra increases were observed in 3D-printed specimens after tea and coffee staining (p < 0.05), but not in artificial saliva (Figure 2). Corega significantly increased Ra in cold-polymerized specimens after artificial saliva immersion, whereas other subgroups showed no significant postcleaning changes (Figure 3).

## 4. Discussion

The optical and surface properties of denture base resins are essential for long-term esthetics, biofilm control, and patient satisfaction. These properties are influenced by fabrication methods, polymerization processes, and material composition, which collectively determine the degree of water sorption, cross-linking, and surface integrity [4,9,20]. In the present study, the color stability and surface roughness of conventionally polymerized (cold- and heat-polymerized) and digitally fabricated (CAD/CAM-milled and 3D-printed) resins were evaluated after immersion in staining beverages (tea, coffee, artificial saliva), followed by cleansing with NaOCl or Corega. The simulated 1 year protocol revealed significant differences between materials and treatments, leading to partial rejection of the null hypotheses.

Discoloration of denture bases may occur through intrinsic and extrinsic mechanisms. Intrinsic changes are associated with water sorption, residual monomer release, and polymer degradation, whereas extrinsic staining arises from dietary chromogens such as polyphenols, tannins, and caffeine [13,24–27]. In the present study, tea produced the most pronounced discoloration in 3D-printed resins, likely due to their layered microstructure and higher surface roughness, which favor pigment adsorption. This finding aligns with Takhtdar et al. [24], who reported lower color stability in 3D-printed resins compared with milled materials. Coffee induced less discoloration in cold-polymerized specimens, possibly due to superficial deposition rather than deep pigment penetration. These material-dependent differences indicate that adsorption predominates in 3D-printed resins, whereas absorption and matrix diffusion may be more relevant in prepolymerized CAD/CAM blocks.

Staining susceptibility was determined not only by fabrication method but also by immersion medium. Consistent with Shin et al. [28], the present study showed that CAD/CAM blocks exhibited greater discoloration in saliva, whereas 3D-printed resins stained more in tea. The acidic pH of tea likely enhanced hydrolysis and pigment uptake in printed resins [14], whereas the higher water sorption and lower cross-link density of CAD/CAM resins may have facilitated diffusion in saliva [7]. These findings emphasize that the clinical risk of discoloration depends not only on resin selection but also on patients’ dietary exposure.

Comparison with earlier studies further highlights this variability. Alp et al. [9] reported imperceptible coffee-related discoloration in CAD/CAM blocks, whereas noticeable changes were observed in the present study, though still within clinically acceptable thresholds. The discrepancy may be linked to preparation methods, immersion conditions, or coffee concentration, reinforcing the role of patient-specific consumption habits. Tieh et al. [29] also observed that saliva and cleansers could cause discoloration comparable to that of pigmented beverages, consistent with the present results. Jain et al. [10] confirmed that intrinsic factors such as water sorption remain key contributors to long-term color instability, particularly in materials with lower polymerization conversion. Collectively, these studies support the conclusion that staining outcomes are influenced by both extrinsic chromogen exposure and intrinsic material characteristics.

Cleaning agents also had significant effects. NaOCl, a strong oxidizing agent, caused greater color change in some subgroups than Corega, consistent with Alqanas et al. [4]. However, under other conditions, no differences were observed, which may reflect variations in material composition and immersion protocols. Alqanas et al. [4] found heat-polymerized resins to be more susceptible to NaOCl-related changes, whereas the present study revealed greater discoloration in 3D-printed resins exposed to both coffee and cleansers. Similarly, Alfouzan et al. [12,17] reported that both NaOCl and effervescent tablets altered surface properties, with greater roughness and discoloration observed in additively manufactured resins. Çakmak et al. [3,16] corroborated these findings, reporting that NaOCl increased surface degradation in printed specimens but had limited effects on CAD/CAM blocks. Collectively, these results indicate that cleaning outcomes depend on material–beverage–cleanser interactions rather than on cleanser type alone.

Baseline roughness patterns further reflected the fabrication techniques. Consistent with previous reports [24], 3D-printed resins exhibited higher Ra values due to additive layering, residual uncured monomers, and greater polarity. Heat-polymerized and CAD/CAM materials generally exhibited smoother surfaces, owing to high-pressure and high-temperature polymerization with fewer voids [2,4]. Cold-polymerized resins displayed higher baseline roughness, attributable to manual mixing and porosity formation [24]. Although all tested materials remained below the 10 μm threshold for clinical unacceptability [30], 3D-printed groups exhibited higher Ra values, which may predispose them to plaque retention and mucosal irritation.

The interaction of staining and cleaning further modulated surface changes. Printed specimens exposed to tea and coffee exhibited significant roughness increases after cleaning, likely due to matrix softening and pigment penetration. Similar observations were made by Çakmak et al. [3,16] and Takhtdar et al. [24], who attributed the vulnerability of printed resins to low polymerization rates and layered structures. Alfouzan et al. [12,17] also reported that Corega altered CAD/CAM resin surfaces, possibly through oxidative and alkaline effects. While NaOCl remains effective in microbial reduction, its bleaching and roughening effects raise esthetic concerns, making effervescent tablets a relatively safer alternative, despite their potential long-term effects.

Clinically, these findings highlight the importance of individualized recommendations. Patients with high tea or coffee consumption may experience earlier discoloration of 3D-printed resins and should be advised to follow stricter hygiene routines or consider alternative material choices. Heat-polymerized and CAD/CAM-milled resins, with their smoother surfaces and lower sorption, appear more favorable for long-term esthetics and plaque control. However, the frequent use of NaOCl should be limited, and effervescent tablets may be preferred, especially for elderly patients with reduced manual dexterity.

The in vitro design cannot fully replicate intraoral conditions, where prostheses are exposed to salivary enzymes, fluctuating pH, mastication, and mechanical brushing. The simulated 1 year aging period does not represent the full clinical lifespan of dentures, which may extend up to 5 years. Furthermore, only selected commercial products and cleanser concentrations were tested, limiting generalizability. Future studies should explore longer aging periods, diverse resin formulations, varied cleanser regimens, and in vivo validation to enhance the clinical relevance of laboratory findings.

## Conclusions

All tested denture base materials exhibited clinically acceptable color changes (ΔE_00_ ≤ 4.1) after a 1 year period of simulated staining and cleaning. Among the cleaning agents tested, Corega provided superior stain removal compared with NaOCl, particularly for heat-polymerized and CAD/CAM-milled materials.

Ra remained below 0.2 μm for heat-polymerized and CAD/CAM-milled resins, indicating a lower risk of plaque accumulation and biofilm retention. In contrast, cold-polymerized and 3D-printed resins exceeded this threshold, especially after exposure to tea and coffee. Artificial saliva had minimal impact on roughness; however, NaOCl increased Ra in certain subgroups, particularly in 3D-printed materials.

## Clinical implications

Clinicians should consider the specific properties of denture base materials when advising patients. Heat-polymerized and CAD/CAM-milled resins are preferable for patients who frequently consume staining beverages, due to their lower susceptibility to discoloration and roughening. Cold-polymerized and 3D-printed resins require more vigilant hygiene monitoring. The use of chemical cleansers, particularly NaOCl, should be carefully managed to minimize long-term surface deterioration. Individualized patient guidance on dietary habits and denture care is essential to preserve esthetics, reduce plaque accumulation, and maintain prosthesis longevity.

## Figures and Tables

**Table 1:** Denture base materials used in this study.

Material	Product	Manufacturer	Polymerization type	Active ingredients
Conventional denture base	Meliodent	Heraeus Kulzer GmbH, Hanau, Germany	Cold-polymerized	PMMA
	Procryla	Vertex Dental BV, Zeist, Netherlands	Heat-polymerized	PMMA
Digital denture base	Yamahachi	Yamahachi Dental MFG, Aichi Prefecture, Japan	CAD/CAM milling	PMMA
	Curo Denture	MACK4D, Ackuretta Technologies Pvt Ltd, Neukieritzsch, Germany	3D printing	Aliphatic difunctional methacrylate, 2,2′-ethylenedioxy diethyl dimethacrylate, aliphatic urethane acrylate

PMMA: poly(methyl methacrylate); CAD/CAM: computer-aided design and computer-aided manufacturing; 3D: three-dimensional

**Table 2:** Devices and instruments used in this study.

Category	Device/Instrument	Manufacturer (City, Country)
CAD software	SolidWorks	Dassault Systemes SolidWorks Corp., Waltham, MA, USA
Milling device	CORiTEC 250i	imes-icore GmbH, Eiterfeld, Hesse, Germany
3D printer	FreeShape 120	Ackuretta Technologies, Taipei, Taiwan
Measuring device	Digital calipers	Mahr GmbH, Göttingen, Germany
Polishing paste	Polishing paste	Ivoclar Vivadent AG, Schaan, Liechtenstein
Colorimeter	Chromameter CR-321	Minolta Co. Ltd., Osaka, Japan
Profilometer	Perthometer	Mahr GmbH, Göttingen, Germany

**Table 3:** Staining and cleaning solutions used in this study.

Category	Product	Manufacturer/Brand	Active ingredient	Preparation
Solutions	Artificial saliva	Prepared in-house (Laboratory mix)	KCl, NaCl, CaCl_2_, NaH_2_PO_4_, Na_2_S, Urea	Components mixed and pH adjusted to 6.5
	Black tea	Lipton Yellow Label, Lipton, Rize, Turkey	Black tea leaves	1 tea bag/200 mL boiling water, steeped for 2 min
	Coffee	Nescafe Classic, Nestle, Bursa, Turkey	Coffee granules	2 g coffee/50 mL boiling water, cooled to room temperature
Cleaning agent	Effervescent tablet (Corega)	Block Drug Company Inc., Jersey City, NJ, USA	Active cleaning tablet	Immersed for 3 min in warm water
	Sodium hypochlorite (NaOCl)	prepared in-house	1% NaOCl (diluted from 5.25% stock solution)	Immersed for 10 min at room temperature

NaCl: sodium chloride; KCl: potassium chloride; CaCl_2_·H_2_O: calcium chloride monohydrate; NaH_2_PO_4_·H_2_O: sodium dihydrogen phosphate monohydrate; Na_2_S: sodium sulfide; NaOH: sodium hydroxide

**Table 4:** Color change (ΔE_00_) values of the test materials after immersion in artificial saliva, tea, and coffee.

ΔE_00_ (t2–t1)	Artificial saliva	Tea	Coffee	X^2^	p
Cold-polymerized	3.86 ± 1.61 3.46 (0.58)^a,A^	3.14 ± 2.23 3.17 (2.51)^a,A,C^	1.71 ± 1.18 1.29 (1.38)^b,A^	18.54	<**0.001**
Heat-polymerized	2.36 ± 1.46 1.98 (1.88)^a,B^	2.03 ± 1.14 1.68 (1.44)^a,B^	4.00 ± 0.56 4.02 (0.96)^b,B^	24.00	<**0.001**
CAD/CAM milling	3.64 ± 0.60 3.74 (0.89)^a,A^	3.08 ± 0.44 3.16 (0.37)^b,A^	2.96 ± 1.02 3.18 (1.30)^a,b,C,D^	9.30	**0.01**
3D printing	1.59 ± 0.61 1.51 (1.18)^a,B^	3.56 ± 0.84 3.51 (0.49)^b,C^	3.57 ± 1.14 3.47 (1.19)^b,B,D^	34.05	<**0.001**
X^2^	41.33	18.03	35.09		
p	<**0.001**	<**0.001**	<**0.001**		

Lowercase letters indicate differences between solutions within the same material (row-wise comparisons).

Capital letters indicate differences between materials within the same solution (column-wise comparisons).

**Table 5:** Statistical comparison of color change (ΔE_00_) values after staining and cleaning across materials.

ΔE_00_ (t3–t2)	Artificial saliva		Tea		Coffee	
	Corega	NaOCl	Corega	NaOCl	Corega	NaOCl
Cold-polymerized	4.22 ± 1.17 3.80 (0.66)^A^	4.11 ± 2.48 3.50 (1.05)^A^	1.91 ± 0.81 1.80 (1.30)^A^	4.16 ± 1.70 3.63 (1.84)^A^	2.19 ± 1.11 1.63 (1.73)^A^	2.28 ± 1.60 1.92 (1.07)^A,B^
Heat-polymerized	1.30 ± 0.54 1.20 (1.07)^B^	3.35 ± 1.65 3.51 (2.41)^A,B^	1.40 ± 0.60 1.50 (0.99)^A^	1.98 ± 0.96 1.92 (0.84)^B^	1.36 ± 0.50 1.23 (0.53)^A^	1.41 ± 0.78 1.24 (0.86)^A^
CAD/CAM milling	1.98 ± 1.21 1.80 (1.98)^B^	1.94 ± 0.60 1.96 (1.03)^B^	1,54 ± 0.24 1.58 (0.44)^A^	2.13 ± 0.47 2.03 (0.89)^B^	1.56 ± 0.50 1.44 (0.85)^A^	1.29 ± 0.33 1.27 (0.57)^A^
3D printing	1.25 ± 0.59 1.14 (0.81)^B^	2.09 ± 0.36 1.99 (0.52)^B^	1.72 ± 0.48 1.63 (0.55)^A^	2.27 ± 0.57 2.11(1.01)^B^	3.14 ± 0.68 3.27 (1.03)^B^	3.45 ± 1.16 3.25 (1.51)^B^
Test statistic	21.064++	16.706++	1.546+	20.221++	11.723+	20.318++
p	<**0.001**	**0.001**	0.219	<**0.001**	<**0.001**	<**0.001**

+ One-way ANOVA;

++ Kruskal–Wallis H test

Capital letters indicate differences between materials within the same solution and cleaning agent (column-wise comparisons)

**Table 6:** Surface roughness (Ra) values of the test materials at baseline, after immersion in solutions, and after cleaning.

			Mean±SD Median (IQR)	Mean±SD Median (IQR)	Mean±SD Median (IQR)	Total	Test statistics/p
Baseline	Cold-polymerized		0.29 ± 0.42 0.18 (0.07)	0.28 ± 0.17 0.21 (0.13)	0.32 ± 0.32 0.22 (0.10)	0.30 ± 0.31 0.21 (0.10)^A^	96.199†
	Heat-polymerized		0.12 ± 0.59 0.10 (0.07)	0.13 ± 0.08 0.10 (0.05)	0.18 ± 0.11 0.15 (0.13)	0.14 ± 0.09 0.10 (0.08)^B^	**<0.001**
	CAD/CAM milling		0.18 ± 0.12 0.12 (0.18)	0.19 ± 0.14 0.12 (0.18)	0.17 ± 0.10 0.13 (0.12)	0.18 ± 0.12 0.12 (0.12)^B^	
	3D printing		0.39 ± 0.18 0.36 (0.25)	0.45 ± 0.22 0.43 (0.43)	0.35 ± 0.16 0.32 (0.22)	0.40 ± 0.19 0.36 (0.27)^C^	
			Artificial saliva	Tea	Coffee	F/X^2^	p
After solutions	Cold-polymerized		0.25 ± 0.56 0.25 (0.09)^a^	0.25 ± 0.07 0.23 (0.09)^a^	0.33 ± 0.28 0.23 (0.09)^a^	0.077†	0.962
	Heat-polymerized		0.14 ± 0.04 0.15 (0.07)^a^	0.11 ± 0.04 0.10 (0.05)^a^	0.15 ± 0.09 0.12 (0.12)^a^	4.562†	0.102
	CAD/CAM milling		0.25 ± 0.12 0.23 (0.22)^a^	0.23 ± 0.13 0.20 (0.13)^a^	0.20 ± 0.09 0.17 (0.11)^a^	1.437†	0.487
	3D printing		0.39 ± 0.18 0.33 (0.27)^a^	0.51 ± 0.26 0.50 (0.44)^a^	0.42 ± 0.1 0.41 (0.19)^a^	2.457†	0.293
			Artificial saliva	Tea	Coffee	F/X^2^	p
After cleaning	Cold-polymerized	Corega	0.48 ± 0.46 0.23 (0.50)^a,1^	0.37 ± 0.25 0.28 (0.19)^a,1^	0.31 ± 0.08 0.28 (0.11)^a^	0.301†	0.860
		NaOCl	0.40 ± 0.56 0.23 (0.09)^a,1^	0.26 ± 0.05 0.26 (0.09)^a,1^	0.40 ± 0.36 0.23 (0.21)^a^	1.104†	0.576
		p	0.471**	0.405**	0.223**		
	Heat-polymerized	Corega	0.11 ± 0.03^a,1^ 0.11 (0.03)	0.13 ± 0.06^a,c^ 0.12 (0.09)^1^	0.18 ± 0.08^b,c^ 0.20 (0.14)	4.445	**0.021** *
		NaOCl	0.14 ± 0.12 0.10 (0.10)^a,1^	0.18 ± 0.10 0.14 (0.08)^a,1^	0.19 ± 0.09 0.20 (0.12)^a^	3.411†	0.182
		p	0.940**	0.239**	0.893+		
	CAD/CAM milling	Corega	0.25 ± 0.18 0.25 (0.34)^a,1^	0.17 ± 0.06^1^ 0.14 (0.09)^a^	0.24 ± 0.19 0.16 (0.25)^a^	0.153†	0.926
		NaOCl	0.15 ± 0.08 0.11 (0.16)^a,1^	0.26 ± 021^1^ 0.17 (0.28)^a^	0.24 ± 0.21 0.15 (0.25)^a^	2.122†	0.346
		p	0.271**	0.225+	0.909**		
	3D printing	Corega	0.34 ± 0.19 0.26 (0.24)^a,1^	0.56 ± 0.35^1^ 0.55 (0.53)^a^	0.41 ± 0.12 0.42 (0.25)^a^	3.674†	0.159
		NaOCl	0.63 ± 0.45 0.43 (0.38)^a,2^	0.59 ± 0.33^1^ 0.46 (0.66)^a^	0.49 ± 0.31 0.37 (0.60)^a^	1.339†	0.512
		p	**0.026** **	0.857+	0.478+		

* p < 0.05;

† : Kruskal–Wallis test;

** Mann–Whitney U test;

+ Independent t-test

Lowercase letters indicate differences between solutions within the same material (row-wise comparisons)

Capital letters indicate differences between materials (column-wise comparisons)

Numbers indicate differences between cleaning agents within the same solution and material (column-wise comparisons)
